# Some Observations Respecting the Structure and Functions of the Udder of the Cow

**Published:** 1821-06

**Authors:** James White


*Some Observations respecting the Structure and Functions of the Udder of the Cow.* By Mr. JAMES WHITE.

THERE is a curious provision in the structure and economy of the udder of the cow, which appears worthy of the attention of physiologists in general. I have shown, in some Papers written for the Bath Agricultural Society in the course of last year, that the cow's udder is not a gland, as it is commonly supposed to be, but merely a receptacle for milk, which is chyle, and is formed in the fourth stomach. This is capable of a clear and simple demonstration. When the cow arrives at a certain age, and begins to approach towards that state when she is no longer capable of breeding or forming milk, there is a provision for a gradual obliteration of the udder; the cells of which it is composed being broken down, and changed into a thick purulent fluid, which may be drawn off from the teat by stroking it. Most commonly, however, it is left entirely to nature, and a gradual thickening and hardening of the quarter, as it is termed, takes place. The pus appears to become solid and organized, or the purulent fluid may in some instances drain off; but in either case the quarter is gradually filled up, and changed into that fatty kind of substance which we observe in the udder of cow-beef.

A similar process (but without any purulent fluid being formed, which happens only, I believe, in old cows,) takes place in the animal at all ages, when kept from breeding; and, when the animal is young and fat, the udder approaches more nearly to the same state,—that is, good fat.

I should have observed, that the cow's udder is composed of four distinct receptacles, which are commonly named quarters, each of which is supplied by a distinct milk vessel. The two trunks of these vessels may be seen under the cow's belly, when the udder is distended with milk; and, if punctured when in this state, the proof I have before alluded to may be obtained.

I have observed, in fat cows, that the teat is converted into fat; but, on cutting off the teat transversely, some mark of the cavity is generally seen.

The discovery I have made of the structure and economy of the cow's udder, and the experience I have had in the diseases of cattle within the few years that I have devoted a considerable portion of my time to the subject, has enabled me, I trust, to make some considerable improvements in this branch of veterinary science. The result of my researches will soon be published, in two volumes: the one, a Compendium of Cattle Medicine; and the other, Essays on the Structure, Economy, and Diseases of Cattle, and especially of the digestive and lactiferous system of the Milch-Cow.

*Bath; April* 5, 1821.

---

